# Assessment of psychological distress and quality of life of family caregivers caring for patients with chronic diseases at home

**DOI:** 10.3934/publichealth.2023032

**Published:** 2023-05-24

**Authors:** Anastasia Stathopoulou, Georgios F. Fragkiadakis

**Affiliations:** 1 Professional Health Worker, Metaxa Cancer Hospital of Piraeus, Athens; 2 Collaborating Teaching Staff, School of Social Sciences, Hellenic Open University, Patra, Greece

**Keywords:** chronic diseases, family caregiver burden, caregiving outcomes, home care

## Abstract

**Introduction:**

Caring for the chronically ill at home is a particularly demanding process that can place a great burden on the caregiver. International and Greek studies underline and confirm this problem. In addition, family caregivers are not supported by the health systems of the different countries, especially in Greece, where the system relies mainly on the family to care for these patients, which is even more challenging during the Covid-19 pandemic.

**Aim:**

The aim of this study is to evaluate the psychological burden of family caregivers of the chronically ill and the outcomes of care for these individuals. It also aims to assess the intensity of burden and changes in quality of life of family caregivers by demographic characteristics.

**Methods:**

The sample of the study was a random sample and consisted of 102 family caregivers of chronically ill patients registered in home care of “Metaxa” hospital. The scales (BAKAS/BCOS) and (HADS) were used for data collection. SPSS 25 statistical package was used for statistical analysis of the results.

**Results:**

The results of the study, calculated with the BCOS scale, show a low burden (−0.93) of family caregivers, patients with chronic diseases and moderate depression and anxiety. The results of the analysis associate the intensity of family caregiver burden with increased levels of anxiety and depression. The factors that affect burden are gender, with women having higher burden, living with the patient, and low education level. According to the HADS anxiety scale, family caregivers had an average score of 11, indicating a moderate level of anxiety, and for depression, the average score was 10.4, also indicating a moderate level of depression. The results indicate that the state needs to support family caregivers and take immediate action to create structures and implement actions to help families continue in their difficult roles in a pain-free manner.

## 1. Introduction

Mortality in the modern world is mainly due to chronic diseases. The increase in life expectancy and the development of technology lead to a significant increase in chronic diseases. According to the data of the Eurostat report [1], the average number of European citizens over 65 is about 97,000,000. Greece is among the three countries with the highest percentage of older people, 21.2%.

Since there are no or minimal care structures for the chronically ill in various countries, we can observe the phenomenon of informal caregivers who provide care out of love, a sense of duty or for social reasons. Usually, these are people from the family environment [2]. Family caregivers take on more and more responsibility and often do not have the necessary knowledge and skills. This situation leads to severe stress that negatively affects their physical, emotional, and mental health [3]–[5].

Several studies [6],[7] highlight the relationship between social support and psychological pressure exerted on caregivers. Caring for a relative is a stressful event with negative health consequences [8]. In the meta-analysis by Del-Pino-Casado et al. [9], which included 56 studies with adult or older caregivers and analyzed the relationship between support and stress, it was found that there was a moderate negative correlation between them. Caregivers' subjective stress is a situation that can threaten their physical and mental health [10]. Subjective perception of stress leads to anxiety and depression [8]. The above conclusions reinforce the policy of interventions that promote social support for caregivers to prevent and alleviate psychological stress.

The aim of this study is to analyse the level of quality of life and health of family caregivers registered in the inpatient clinic of the Cancer Hospital “METAXA” with chronic and intractable diseases. The main research questions of the study were the following. First, to determine the extent of psychological distress experienced by family caregivers of chronically ill patients. Second, to identify factors associated with or influencing the psychological distress of family caregivers based on specific characteristics. Third, to identify the main problems of family caregivers in relation to their health and quality of life. In Greece, there are not many studies on the burden of family caregivers of patients with chronic diseases.

## 2. Materials and study sample

The study population consisted of the family caregivers of patients with chronic diseases registered with the home care service of the hospital “Metaxa”. The individuals/family caregivers included in the study were selected according to certain criteria related to the following:

a. they had to provide assistance without financial remuneration,

b. they had to be the main caregiver of the patient,

c. they had to have a very good command of the Greek language, and

d. they could not have been diagnosed with a psychiatric illness.

Some criteria (unpaid care, main caregiver) were also used in other studies [6].

To participate in the study, they had to sign an informed consent form. Along with the questionnaires, family caregivers received an information sheet explaining the purpose of the study and the non-binding nature of their participation in the study. They were given the opportunity to ask and answer questions about the study. One hundred and two family caregivers who agreed to participate made up the sample for this study. The sample of the study was a random sample selected from the patients of the home clinic of the hospital “Metaxa”. After signing the informed consent form, the family caregivers completed the questionnaires. The Board of Directors of Metaxa Hospital approved this study on 8/1/2021.

In the present study, the Hospital Anxiety and Depression Scale (HADS) and the Bakas Caregiving Outcomes Scale (BCOS) were used to assess caregiver anxiety and depression. The questionnaire (HADS) was developed by Zigmond & Snaith [11] to assess anxiety and depression in hospitalised patients. It is a widely used questionnaire for many categories of patients and their caregivers [12]. The HADS scale consists of two subscales-HADS-A, which measures anxiety with seven questions, and HADS-D, which measures depression with another seven questions-and is scored separately. Each item is answered with a score of 0–3, so the expected scores for each category are 0–21. A score of 0–7 is normal, 8–10 mild, 11–14 moderate, and 15–21 severe. The HADS scale was translated and validated in Greek by previous study [13].

The Bakas Caregiving Outcomes Scale (BCOS) captures caregivers' assessment of how much their lives have changed since they assumed responsibility for the patient. The revised BCOS with 15 questions was used for the study. Validity questions are scored on a scale of −3 (changed for the worse) to +3 (changed for the better). Higher scores reflect more positive outcomes for the caregiver. The original BCOS was developed and tested based on a similar model that had been used to find evidence of emotional distress, general health beliefs, and consequences of caregiving after stroke [14],[15]. In Greece, the translation of the revised BCOS instrument was validated by testing it on a group of family caregivers of terminal cancer patients undergoing radiation [16]. The SPSS 25 statistical package was used for statistical analysis of the results.

## 3. Results

Results on the demographic characteristics of the 102 family caregivers of chronically ill patients who participated in the survey are shown in Table 1. From the analysis, 68.6% (n = 70) of the sample were female and 31.4% (n = 32) were male. Regarding the educational level of the family caregivers, it was found that 4.9% (n = 5) had not attended school, 23.5% (n = 24) had completed elementary school, 48.1% (n = 49) were high school graduates, and 23.5% (n = 24) had a university education. Table 1 also shows the results in terms of age, years of engagement with patient, the degree of affinity with the patient and hours worked per day by family caregivers. The average age of the sample was 53.7 (SD = 10.9) years, with an age distribution ranging from 24 to 86 years. Family caregivers worked with the patient for an average of 6.8 years (SD = 9.9). They also worked an average of 8.7 (SD = 1.2) hours per day.

Table 2 shows the results regarding whether family caregivers have health problems. The analysis showed that 59.8% (n = 61) of family caregivers had one or more health problems. Of these 61 family caregivers, 34 (33.3%) had musculoskeletal problems, 24 (23.5%) had hypertension, and 15 (14.7%) had cardiovascular disease.

The following Table 3 contains information about the patients. The data presented can further explain the results of the study in the next section. These data refer to the average age of the patients by sex, which is about 77 years for both sexes. In addition, the study shows that the majority of patients (84 out of 102) have difficulties in their daily activities, such as bathing and using the toilet, eating, dressing or preparing for sleep. The above-mentioned functional limitations are a consequence of the chronic diseases described in the same table and mainly concern, 72%, cancer.

**Table 1.** Demographic characteristics of the sample.

		ν	%
Gender	Male	32	31.4%
	Female	70	68.6%
Family status	Married	68	66.7%
	Single	16	15.7%
	Widowed	9	8.8%
	Divorced	9	8.8%
Number of Children	1	21	20.6%
	2	47	46.0%
	3	6	5.9%
	4	5	4.9%
Educational Level	Not attended School	5	4.9%
	Primary School	24	23.5%
	Secondary School	49	48.1%
	Higher Education / University	24	23.5%
Profession	Unskilled worker	5	4.9%
	Skilled worker	2	1.9%
	Freelance	18	17.6%
	Farmer	2	1.9%
	Private sector employee	24	23.5%
	Civil servant	21	20.6%
	Housekeeper	30	29.6%
Place of residence	Same home as the patient	42	41.2%
	Same building as the patient	21	20.6%
	Same neighborhood as the patient	9	8.8%
	Different house and neighborhood	30	29,4%
Consanguinity/ Affinity with the patient	Spouse	16	15.6%
	Daughter	41	40.2%
	Son	17	16.6%
	Sister	4	4.0%
	Brother	3	3.0%
	Other	22	20.6%

**Table:** 

Caregiver Characteristics	Average	Standard Deviation (SD)	Minimum	Maximum
Age	53.7	10.9	24.0	86.0
Number of family members	3.0	1.2	1.0	6.0
Years of patient engagement	6.8	9.9	0.5	50
Working hours	8.7	1.2	7.0	12.0

**Table 2.** Health status results of family caregivers.

		ν	%
Health problems of family caregivers	No problem	41	40.2%
	Some kind of problem	61	59.8%
Type of health problem	Hypertension	24	23.5%
	Diabetes	7	6.9%
	Musculoskeletal	34	33.3%
	Cardiovascular	15	14.7%
	Vision problems	8	7.8%
	Hearing problems	5	4.9%
	Other	6	5.9%

**Table 3.** Patient information and health status.

Patient's Characteristics	ν	Average Age (min.–max.)
Male patients	40	77.47 (57.0–96.0)
Female patients	62	77.15 (28.0–93.0)
Difficulties on Activities of Daily Living (ADLs)	Yes: 84 No: 18	
Type Of Chronic Illness	ν	%
Myelodysplastic Syndrome (MDS)	38	37%
Lymphoma, Multiple Μyeloma (MM), CA Prostate, CA Breast, CA Lung, CA Ovary	36	35%
Chronic Lymphocytic Leukemia (CLL), Acute Μyeloid Leukemia (AML), Chronic Μyelogenous Leukemia (CML), Acute Lymphocytic Leukemia (ALL)	15	15%
Other (Thrombocytopenia, Central Pontine Myelinolysis-CPM, Thrombosis, Anemia, Μalignant Αnemia, Chronic Respiratory Failure)	13	13%

### 3.1. Results (BCOS) on burden level

This section presents the results of the analysis related to changes in family caregivers' lives and their stress levels. Table 4 shows that caring for patients with chronic illnesses stressed family caregivers mainly in terms of their ability to cope with stress (MT = −1.5, TA = 0.8), their emotional well-being (MT = −1.5, TA = 0.9), their time for social activities with friends (MT = −1.5, TA = 0.9), and their time for family activities (MT = −1.4, TA = 0.9). In addition, caregivers recognized that their lives had changed for the worse as a result of caring for the patient (MT = −1.5, TA = 0.8).

Conclusions for the total scale burden (BCOS) showed that the mean value of the BCOS scale was −0.93 (SD = 0.69), indicating that caring for patients with chronic diseases burdens their family caregivers on average, but to a lesser degree. From the distribution of the total scale, it appears that the majority of family caregivers had an average score between −1 and 0, while some had higher levels of burden (scores below −1). It is also noteworthy that very few family caregivers had a positive average score.

**Table 4.** Factor burdens of BCOS caregiving outcomes scale items.

	Mean	SD	Min	Max
1. My self-esteem	−0.2	1.2	−3	3
2. My physical health	−0.9	1.0	−3	0
3. My time for family activities	−1.4	0.9	−3	2
4. My ability to cope with stress	−1.5	0.8	−3	0
5. My relationship with friends	−1.1	1.0	−3	2
6. My future outlook	−1.0	1.1	−3	0
7. My level of energy	−1.0	1.0	−3	0
8. My emotional well-being	−1.5	0.9	−3	2
9. My roles in life	−0.6	1.2	−3	3
10. My time for social activities with friends	−1.5	0.9	−3	0
11. My relationship with my family	−0.4	1.2	−3	3
12. My financial well-being	−1.0	1.0	−3	0
13. My relationship with the patient	0.6	1.4	−3	3
14. My physical functioning	−0.9	0.9	−3	0
15. My general health	−0.8	0.9	−3	0
16. In general, how has your life change as a result of taking care of the patient	−1.5	0.8	−3	0

### 3.2. Load differences by demographic characteristics and relationship to the patient

Next, the results of the comparisons of the degree of stress in relation to demographic characteristics and in relation to the patient are presented. For this purpose, the t-test and one-way method ANOVA were used. Table 5, shows that the burden level of family caregivers differed to a statistically significant extent in relation to gender (t = 2.494, p = 0.014), place of residence (F = 4.710, p = 0.004), educational level (F = 5.345, p = 0.002), personal health problems (t = 2.907, p = 0.004), and the presence of help with patient care (t = 2.531, p = 0.013). The analysis showed that women (MT = −1.0, TA = 0.7) were more burdened than men (MT = −0.7, TA = 0.7). In addition, family caregivers who lived in the same house as the patient (MT = −1.2, TA = 0.7) were found to be more burdened, while the least burden was observed in family caregivers who lived in a different house and neighborhood (MT = −0.6, TA = 0.6). In terms of educational level, family caregivers who did not go to school (MT = −1.4, TA = 0.7) and elementary school graduates (MT = −1.3, TA = 0.9) were found to have higher levels of burden than middle/high school graduates (MT = −0.7, TA = 0.5) and high school graduates (MT = −0.9, TA = 0.7). The analysis showed that family caregivers with a health problem (MT = −1.1, TA = 0.7) had higher levels of strain than family caregivers who were healthy (MT = −0.7, TA = 0.6).

**Table 5.** Results of the t-test and one-way ANOVA for the degree of burden on family caregiver characteristics.

		Burden		t / F	p
		Mean	SD		
Gender	Male	−0.7	0.7	2.494^t^	0.014
	Female	−1.0	0.7		
Family status	Married	−0.9	0.6	0.474^F^	0.701
	Single	−0.9	0.8		
	Widowed	−1.2	0.8		
	Divorced	−1.0	1.0		
Place of residence	Same household as the patient	−1.2	0.7	4.710^F^	0.004
	Same building as the patient	−0.9	0.6		
	Same neighborhood as the patient	−0.7	0.5		
	Different household and neighborhood	−0.6	0.6		
Educational Level	Not attended School	−1.4	0.7	5.345^F^	0.002
	Primary School	−1.3	0.9		
	Secondary School	−0.7	0.5		
	Higher Education / University	−0.9	0.7		
Annual income	None	−1.0	0.7	0.271^F^	0.846
	5.000 to 10.000 Euros	−0.9	0.7		
	10.000 to 20.000 Euros	−0.9	0.7		
	Over 20.000 Euros	−1.1	0.8		
Health Problems	None	−0.7	0.6	2.907^t^	0.004
	Some problems	−1.1	0.7		
Help from family members	Yes	−0.8	0.6	2.531^t^	0.031
	No	−1.1	0.7		

*Note: t = t-test, F = ANOVA.

### 3.3. Results on the level of anxiety and depression of family caregivers using the scale (HADS)

Table 6 shows that an average score between 1 and 2 was obtained for all questions related to anxiety symptoms, indicating that family caregivers of patients with chronic diseases have moderate anxiety symptoms. Of the symptoms reported, the most common are that family caregivers feel anxious, as if something scary is going to happen (MT = 1.8, TA = 0.8), that they cannot sit comfortably and relax (MT = 1.8, TA = 0.6), and that anxious thoughts run through their minds (MT = 1.7, TA = 0.9).

Table 7 shows that a mean score between 1 and 2 was found for all questions about depression and anxiety symptoms, indicating that family caregivers of patients with chronic diseases have moderately depressive symptoms. Of the symptoms reported, the most common are that family caregivers do not often feel relaxed (MT = 1.6, TA = 0.7), do not look forward to things with joy (MT = 1.6, TA = 0.7), and still do not enjoy the things they used to enjoy (MT = 1.5, TA = 0.7).

**Table 6.** Descriptive results for HADS questions on anxiety.

	Mean	SD	Min	Max
I feel tense or wound up	1.6	0.9	0	3
I get a sort of frightened feeling as if something awful is about to happen	1.8	0.8	0	3
Worrying thoughts go through my mind	1.7	0.9	0	3
I can sit at ease and feel relaxed	1.8	0.6	1	3
I get a sort of frightened feeling like ‘butterflies' in the stomach	1.3	0.7	0	3
I feel restless as I have to be on the move	1.5	0.7	0	3
I get sudden feelings of panic	1.2	0.7	0	3

**Table 7.** Descriptive results for the questions of the HADS scale on depression.

	Mean	SD	Min	Max
I still enjoy the things I used to enjoy	1.5	0.7	0	3
I can laugh and see the funny side of things	1.5	0.7	0	3
I feel cheerful	1.6	0.7	0	3
I feel as if I am slowed down	1.4	0.7	0	3
I have lost interest in my appearance	1.3	0.8	0	3
I look forward with enjoyment to things	1.6	0.7	0	3
I can enjoy a good book or radio or TV program	1.5	0.8	0	3

Next, the results of the comparisons of anxiety and depression scores in relation to demographic characteristics and in relation to the patient are presented. For this purpose, the t-test and a one-tailed ANOVA were used. Table 8 shows that the stress level of family caregivers differed statistically significantly according to gender (t = −5.356, p = 0.000), marital status (F = 2.807, p = 0.044) and educational level (F = 3.935, p = 0.011). The analysis showed that women (MT = 12.3, TA = 3.3) have higher levels of anxiety than men (MT = 8.3, TA = 4.0). In terms of educational level, the analysis revealed that family caregivers who did not go to school (MT = 14.5, TA = 2.6) and elementary school graduates (MT = 12.8, TA = 4.3) have higher levels of stress than middle or high school graduates (MT = 10.6, TA = 3.5) and those who completed higher education (MT = 9.6, TA = 4.2).

The results of the study also showed that the level of depression among family caregivers varied to a statistically significant extent in relation to gender (t = −3.941, p = 0.000), marital status (F = 4.461, p = 0.006), educational level (F = 8.339, p = 0.000), presence or absence of a health problem (t = −2.217, p = 0.029), and involvement of others in patient care (t = −3.142, p = 0.000) differed (Table 9). The analysis showed that women (MT = 11.4, TA = 3.2) had higher levels of depression than men (MT = 8.4, TA = 3.9). From the analysis, family caregivers with a health problem (MT = 11.1, TA = 7.7) had higher levels of depression than family caregivers who were healthy (MT = 9.5, TA = 3.6).

**Table 8.** Results of t-test and one-way ANOVA results for anxiety level by family caregiver characteristics.

		Anxiety		t / F	p
		Mean	SD		
Gender	Male	8.3	4.0	−5.356^t^	0.000
	Female	12.3	3.3		
Family status	Married	11.0	3.8	2.807^F^	0.044
	Single	9.1	4.7		
	Widowed	13.4	3.1		
	Divorced	12.3	4.2		
Place of residence	Same household as the patient	11.7	4.4	0.793^F^	0.500
	Same building as the patient	11.3	3.6		
	Same neighborhood as the patient	11.1	3.5		
	Different household and neighborhood	10.2	3.8		
Educational Level	Not attended School	14.5	2.6	3.935^F^	0.011
	Primary School	12.8	4.3		
	Secondary School	10.6	3.5		
	Higher Education / University	9.6	4.2		
Annual income	None	11.6	3.3	0.357^F^	0.784
	5.000 to 10.000 Euros	10.9	4.2		
	10.000 to 20.000 Euros	10.9	4.2		
	Over 20.000 Euros	10.9	4.9		
Health Problems	None	10.4	4.4	−1.309^t^	0.194
	Some problems	11.5	3.7		
Help from family members	Yes	10.5	3.9	−1.478^t^	0.143
	No	11.7	4.1		

*Note: t = t-test, F = ANOVA.

**Table 9.** Results of t-test and one-way ANOVA for level of depression by family caregiver characteristics.

		Depression		t / F	p
		Mean	SD		
Gender	Male	8.4	3.9	−3.941^t^	0.000
	Female	11.4	3.2		
Family status	Married	10.1	3.5	4.461^F^	0.006
	Single	9.3	3.5		
	Widowed	14.1	2.5		
	Divorced	11.7	4.4		
Place of residence	Same household as the patient	11.6	3.8	2.378^F^	0.075
	Same building as the patient	10.1	3.2		
	Same neighborhood as the patient	9.0	3.4		
	Different household and neighborhood	9.8	3.4		
Educational Level	Not attended School	15.3	2.1	8.339^F^	0.000
	Primary School	12.5	4.5		
	Secondary School	9.3	2.6		
	Higher Education / University	9.5	3.5		
Annual income	None	11.2	3.7	0.185^F^	0.907
	5.000 to 10.000 Euros	10.3	3.7		
	10.000 to 20.000 Euros	10.2	3.6		
	Over 20.000 Euros	10.9	4.5		
Health Problems	None	9.5	3.6	−2.217^t^	0.029
	Some problems	11.1	3.7		
Help from family members	Yes	9.4	3.3	−3.142^t^	0.000
	No	11.6	3.8		

## 4. Discussion

Regarding the changes that have occurred in family caregivers' lives as a result of caregiving, the BCOS research tool concludes that their lives have changed for the worse, but only slightly. It has worsened in terms of their health, personal time for activities with family and friends, and stress management. Greater burden is experienced by women, people with the lowest levels of education, people with health problems, people without caregiving support, and people living in the same house as the patient. In addition, there was no increase in burden depending on age, number of children, number of family members, hours worked, and years of caregiving.

Regarding the level of anxiety and depression measured by the HADS scale, a moderate level of anxiety was observed among family caregivers, with women being more affected than men and those with the lowest level of education being among the most educated. Moderate depression was also observed, which was more strongly related to female gender, lower educational level, and personal health problems of the family caregiver. According to the results, anxiety and depression did not seem to be related to the family caregiver's age, working hours, number of family members, and duration of caregiving tasks.

Research shows that the greater the burden, the more anxiety and depression increase, which is also confirmed by other the studies [17]–[21]. The worst position of the female gender is also confirmed by other studies which concluded that women were more associated with increased anxiety and depression [22],[23].

Madruga et al. [24] argue that, in addition to the relationship between gender and educational level in increasing anxiety and depression, the presence or absence of family caregiver support also plays an important role, which is consistent with the findings of the present study.

In the present study, a large percentage of family caregivers (59.8%) suffered from a chronic health problem. Similarly, the study by Taşdelen & Ateş [25] concluded that half of the family caregivers of patients with a long-term illness also suffered from a chronic condition. Impressively, the Swedish study by Ekström et al. [26] concluded that Swedish family caregivers, especially Swedish women, suffered from increased anxiety and depression despite the introduction of formal patient care in that country. Another study by Talarico et al. [27] examined the different dimensions of living with a patient with Behcet's syndrome (BS) and concluded that a rare disease affects not only the patient himself, but also those who live with him and/or contribute to his care, i.e. his informal caregivers. According to this study, caregivers play an important role in the lives of people with a chronic disease, especially when it is a rare disease. The primary objectives of this study were to explore the perspectives and opinions of caregivers of patients with Behcet's syndrome (BS) and to explore the level of awareness of the disease and the potential impact of BS on the lives of caregivers.

Limitations of the study included the fact that participation was voluntary and several family caregivers did not agree to participate in the study, while several others resigned from their original positions. Many of them were afraid to participate in the survey because they feared that they would lose home health care support if they made financial disclosures or had someone else help them.

## 5. Conclusions

Chronic diseases have increased along with life expectancy, creating a community of patients with chronic illnesses. This situation has led to an increase in the number of caregivers and the phenomenon of atypical caregivers, mainly from the family. The aim of this study was to identify and measure the burden of family caregivers of patients with chronic diseases in home care at Metaxa Hospital in relation to various parameters. The results of the study show that family caregivers experience a burden when caring for patients with severe chronic diseases. The main impacts on participants' lives were emotional well-being, ability to manage stress, time for social activities with friends and time for family activities. Place of residence also appeared to be related to increased stress, for those who lived in the same house. Another aggravating factor was education level, as those with a low level of education experienced stress more intensely. In addition, the most common symptoms suffered by family caregivers were anxiety and mild depression.

We believe that future research could shed light on how caring for relatives triggers anxiety and depression and suggest specific interventions that can be used to support caregivers. Research such as this helps to highlight the consequences of informal caregiving, health and mortality, and may help to redesign family caregiving programs.

## References

[1] Eurostat (2020). Statistics Explained. Population structure and ageing.

[2] Ahrens J, Nadash P (2003). Informal caregivers: who are they & how do they differ?. Caring: Natl Assoc for Home Care mag.

[3] Pinquart M, Sörensen S (2003). Differences between caregivers and noncaregivers in psychological health and physical health: A meta-analysis. Psychol Aging.

[4] Schulz R, Sherwood PR (2008). Physical and mental health effects of family caregiving. Am J Nurs.

[5] Vitaliano PP, Zhang J, Scanlan JM (2003). Is caregiving hazardous to one's physical health? A meta-analysis. Psychol Bull.

[6] Wang HH, Wu SZ, Liu YY (2003). Association between social support and health outcomes: a meta-analysis. Kaohsiung J Med Sci.

[7] Holt-Lunstad J, Smith TB, Layton JB (2010). Social Relationships and Mortality Risk: A Meta-analytic Review. PLoS Med.

[8] Pinquart M, Sörensen S (2003). Differences between caregivers and noncaregivers in psychological health and physical health: a meta-analysis. Psychol Aging.

[9] Del-Pino-Casado R, Priego-Cubero E, López-Martínez C (2021). Subjective caregiver burden and anxiety in informal caregivers: A systematic review and meta-analysis. PloS One.

[10] Kim H, Chang M, Rose K (2012). Predictors of caregiver burden in caregivers of individuals with dementia. J Adv Nurs.

[11] Zigmond AS, Snaith RP (1983). The hospital anxiety and depression scale. Acta Psychiat Scand.

[12] Litster B, Fiest KM, Patten SB (2016). “Defining the Burden and Managing the Effects of Psychiatric Comorbidity in Chronic Immunoinflammatory Disease”. Screening Tools for Anxiety in People with Multiple Sclerosis: A Systematic Review. Int J MS care.

[13] Mystakidou K, Tsilika E, Parpa E (2004). The Hospital Anxiety and Depression Scale in Greek cancer patients: psychometric analyses and applicability. Support Care Cancer.

[14] Bakas T, Champion V (1999). Development and psychometric testing of the Bakas Caregiving Outcomes Scale. Nurs Res.

[15] Bakas T, Burgener SC (2002). Predictors of emotional distress, general health, and caregiving outcomes in family caregivers of stroke survivors. Top Stroke Rehabil.

[16] Govina O, Kotronoulas G, Mystakidou K (2013). Validation of the revised Bakas Caregiving Outcomes Scale in Greek caregivers of patients with advanced cancer receiving palliative radiotherapy. Support Care Cancer.

[17] Timonet-Andreu E, Morales-Asencio JM, Alcalá Gutierrez P (2020). Health-Related Quality of Life and Use of Hospital Services by Patients with Heart Failure and Their Family Caregivers: A Multicenter Case-Control Study. J Nurs Scholarship.

[18] Kusi G, Boamah Mensah AB, Boamah Mensah K (2020). The experiences of family caregivers living with breast cancer patients in low-and middle-income countries: a systematic review. Syst Rev.

[19] Paschou A, Damigos D, Skapinakis P (2018). The Relationship between Burden and Depression in Spouses of Chronic Kidney Disease Patients. Depress Res Treat.

[20] Greco A, Pancani L, Sala M (2017). Psychometric characteristics of the caregiver burden inventory in caregivers of adults with heart failure. Eur J Cardiovasc Nur.

[21] Bhimani R (2014). Understanding the Burden on Caregivers of People with Parkinson's: A Scoping Review of the Literature. Rehabil Res Pract.

[22] Vrettos I, Kamposioras K, Kontodimopoulos N (2012). Comparing health-related quality of life of cancer patients under chemotherapy and of their caregivers. Sci World J.

[23] Andreakou MI, Papadopoulos AA, Panagiotakos DB (2016). Assessment of Health-Related Quality of Life for Caregivers of Alzheimer's Disease Patients. Int J Alzheimers Dis.

[24] Madruga M, Gozalo M, Prieto J (2020). Psychological Symptomatology in Informal Caregivers of Persons with Dementia: Influences on Health-Related Quality of Life. Int J Env Res Pub health.

[25] Taşdelen P, Ateş M (2012). Evaluation of the burden of caregivers and home care needs of patients requiring care. J Nurs Res Dev.

[26] Ekström H, Auoja NL, Elmståhl S (2020). High Burden among Older Family Caregivers is Associated with High Prevalence of Symptoms: Data from the Swedish Study “Good Aging in Skåne (GÅS)”. J Aging Res.

[27] Talarico R, Marinello D, Manzo A (2021). Being a caregiver of a Behçet's syndrome patient: challenges and perspectives during a complex journey. Orphanet J Rare Dis.

